# Evaluation of antibacterial agent use in adult patients at a tertiary-level hospital in Jordan: A comparative analysis of defined daily dose and days of therapy in critical care units

**DOI:** 10.1371/journal.pone.0356239

**Published:** 2026-08-20

**Authors:** Amjad Bani Hani, Walid Al-Qerem, Walaa Ashran, Farah Bani Hani, Sara Al-Raymoony, Salma Elshiekh, Miramar Haddad, Yarub Alsheyab, Ghada Alzyadat, Enas Khasawneh, Ali Heis, Tarek Gharibeh, Raghad Al-Shweiki, Awni D. Shahait

**Affiliations:** 1 Department of General Surgery, Faculty of Medicine, The University of Jordan, Amman, Jordan; 2 Department of Pharmacy, Faculty of Pharmacy, Al-Zaytoonah University of Jordan, Amman, Jordan; 3 Department of Clinical Pharmacy, Jordan University Hospital, The University of Jordan, Amman, Jordan; 4 Faculty of Medicine, The University of Jordan, Amman, Jordan; 5 Department of Anaesthesiology, Royal Medical Services, Amman, Jordan; 6 Public Health Institute, Faculty of Medicine, The University of Jordan, Amman, Jordan; 7 Department of Surgery, Southern Illinois University School of Medicine, Carbondale, Illinois, United States of America; Universidad de Murcia Faculty of Economics and Business: Universidad de Murcia Facultad de Economia y Empresa, SPAIN

## Abstract

**Objective:**

To compare two measures of antibiotic stewardship: Defined Daily Dose (DDD) versus Days of Therapy (DOT).

**Methods:**

Retrospective observational analysis of clinical data for adult ICU patients with antimicrobial exposure recorded from July 2023 to July 2024. The variables collected were the DDD and DOT. The DDD variable was defined according to the World Health Organization (WHO) standards for all antibacterial medications. For the DOT variable, DOTs reflected each day a drug was given, regardless of dose count. For comparability, total DDDs and DOTs were standardized per 100 patient-days using the 6,614 patient-days accrued by the antimicrobial-exposed ICU cohort as the denominator.

**Results:**

Over the study duration, 1,024 patients were admitted to adult ICUs, of whom 607 received antimicrobial agents. The overall median total DDD per antimicrobial agent was 64.3 (interquartile range [IQR]: 6.6–530.0), while the corresponding median total DOT was 47.0 (IQR: 6.0–581.5), reflecting considerable variation in absolute antimicrobial measurement across the two methods (Wilcoxon signed-rank test across patient-antimicrobial pairs: V = 130,979, p < 0.001).

**Conclusions:**

DDD and DOT remained strongly correlated but showed clinically meaningful drug-level disagreement in this ICU cohort. DOT provides a clinically interpretable measure of antimicrobial exposure duration in ICU patients because it reflects treatment days irrespective of dose, whereas DDD remains useful for standardized benchmarking across institutions and time periods. The two measures provide complementary information on antimicrobial use and should not be interpreted as substitutes for one another.

## Introduction

Antimicrobial stewardship programs (ASPs) have become increasingly important in healthcare settings, as they aim to optimize antimicrobial use and minimize the development of resistance [1]. Understanding antibiotic prescribing patterns in hospitals is essential for combating antibiotic resistance [2,3]. One of the most widely recognized and adopted measures of antimicrobial usage is the defined daily dose (DDD), which allows researchers to standardize the quantity of medication utilized and acquisition costs per admission [4].

The DDD, as defined by WHO, represents the average maintenance dose per day assumed for the drug’s primary approved use in adult patients and is useful for comparing patterns of antibiotic use across hospitals or countries. However, some issues with DDD include discrepancies between assigned and actual doses, underestimation in renal impairment, and lack of assigned DDDs for pediatric populations [5].

Antibiotic utilization can be measured using days of therapy (DOT) by counting the total treatment days with an antibiotic, irrespective of the administered dosage. This approach has been recommended by both the Infectious Diseases Society of America (IDSA) and the Society for Healthcare Epidemiology of America (SHEA). The DOT method can also be applied to paediatric populations, although obtaining patient-level data can be challenging in some facilities [6,7].

Evaluating antimicrobial consumption within the intensive care unit (ICU) is crucial for antimicrobial stewardship programs, infection control, and understanding resistance patterns [8]. The best way to measure antibiotic usage is still under debate [9]. In intensive care units, DDD may be inappropriate among critically ill patients because changes in physiological parameters can significantly affect the pharmacokinetics of antimicrobials, necessitating adjustments to the administered doses. Therefore, application of the DDD method can lead to errors in measuring the patterns of antibiotic use in ICUs. On the other hand, the DOT method is easier to implement in environments where prescriptions are recorded at the individual-patient level [9–11]. However, the DOT method is not without constraints. One challenge is the complexity of critically ill patients receiving multiple antimicrobial agents concurrently.

We conducted this study to assess the total antibiotic consumption in an intensive care unit (ICU) at a university hospital, employing both the DDD and DOT methods in order to compare these metrics and confirm the discrepancies between the two methodologies. We hypothesized that the DDD method overestimates antibiotic consumption, especially in ICU patients, due to the disparities between DDD and the dosages of antimicrobials administered to critically ill patients [9].

## Methods

### Study design and population

The study was conducted retrospectively at a tertiary academic hospital in Jordan. Data were collected on antimicrobial usage for adult patients admitted to the ICU with antibiotic exposure recorded from July 2023 to July 2024. Clinical records were accessed and data were extracted between May 1 and August 16, 2025, after institutional review board approval had been obtained in April 2025. Data analysis was conducted in January 2026.

### Ethical approval

This study was approved by the Institutional Review Board of Jordan University Hospital in April 2025 (Approval No. 10/2025/9204). Patients provided general admission consent for the use of de-identified medical-record data in ethically approved research; no additional study-specific consent was obtained retrospectively.

### Patient demographic data

Relevant information, including patient age, gender, length of stay, comorbidities, and prescribed antibacterial drugs, was collected.

### Antibacterial drug data collection

Data on antibacterial drugs administered to patients were collected by trained physicians. The names of the antibacterial drugs, the dates of administration, the doses administered, and the routes of administration were collected using the daily records in the pharmacy department’s electronic database for patients admitted to the adult ICU.

The Defined Daily Dose (DDD) is a standardized metric established by the World Health Organization (WHO) Collaborating Centre for Drug Statistics Methodology. It represents the assumed average maintenance dose per day for a drug used for its primary indication in adults. This unit facilitates consistent comparisons of drug consumption across different settings and time periods [5].

For each administered antibacterial agent, the corresponding DDD values were sourced from the 2025 WHO Anatomical Therapeutic Chemical (ATC) Classification System. The total number of DDDs was calculated as the total amount of drug used in the WHO-assigned unit divided by the WHO-assigned DDD for the relevant route and unit. For most antimicrobial agents, this unit was grams per day; for colistin, the WHO DDD unit was million units (MU). This approach enables the quantification of antimicrobial use in a standardized manner, allowing for monitoring and comparison of antibiotic consumption patterns. Antibiotic consumption was calculated in DDDs per 100 patient-days to standardize aggregate use [9].

Days of Therapy (DOT) was defined as the number of calendar days on which a patient received a given antimicrobial agent, irrespective of the number of doses or dosage strength administered. If a patient received more than one antimicrobial on the same day, each antimicrobial contributed one DOT [12]. Total DOT was standardized per 100 patient-days. For extended-interval regimens, including renal-adjusted levofloxacin administered every other day, the primary analysis counted DOT on actual administration days [13]; an alternative therapeutic-coverage DOT definition was assessed in the sensitivity analysis. Dosing of certain drugs was not uniform across patients and was adjusted based on therapeutic drug monitoring of blood levels due to changes in pharmacokinetics in critical care settings, such as low creatinine clearance and increased volume of distribution [14].

### Statistical analysis

For each antimicrobial agent, DDD and DOT were calculated at the patient-antimicrobial-record level and then aggregated across all patients receiving the same drug. Standardized rates were calculated by dividing each drug-specific DDD and DOT total by the total available ICU patient-days in the antimicrobial-exposed cohort (6,614 patient-days) and multiplying by 100. The percentage difference was calculated as (DDD – DOT) / DDD x 100 and classified as minor (<5%), moderate (5–25%), or major (>25%). The administered daily dose was calculated by multiplying the prescribed frequency by the recorded dose in the unit used for the WHO DDD. For non-colistin agents, this unit was grams per day. Colistin was analyzed in million units (MU), using WHO route-specific DDD values of 9 MU for parenteral records and 3 MU for nebulized records. These cohort-based rates are not directly comparable with whole-ICU consumption rates calculated using all ICU patient-days.

For the paired comparison, repeated records for the same patient and antimicrobial agent were summed first, producing one paired DDD and DOT value per patient-antimicrobial combination. Patients receiving more than one antimicrobial contributed separate patient-antimicrobial pairs because DDD and DOT are drug-specific exposure measures. The Wilcoxon signed-rank test was then used to compare paired DDD and DOT totals across patient-antimicrobial pairs because the distribution of paired differences was non-normal. Agreement between drug-level standardized DDD and DOT rates was additionally assessed using a Bland-Altman approach based on the mean of the two rates and their difference. For extended-interval regimens, a sensitivity analysis used an alternative therapeutic-coverage DOT definition rather than the primary actual-administration DOT definition; patient-antimicrobial DOT values were capped at ICU length of stay. This alternative DOT definition was applied to the drug-level standardized rates, regression and agreement analyses, and paired Wilcoxon comparison. Statistical significance was determined at p < 0.05. Statistical analyses were conducted using R version 4.5.3. Detailed calculation definitions and verification outputs for DDD, DOT, standardized rates, percentage differences, and statistical summaries are provided in S1 Appendix.

## Results

The study reviewed the records of 1,024 patients, of whom 607 received antimicrobial treatment and were included in the final dataset. This antimicrobial-exposed ICU cohort represented 6,614 patient-days. The participants had a mean age of 64.1 years (standard deviation [SD] = 18.8). Males comprised 55% of the sample. Most participants were not medically free (82%), whereas 18% had no recorded comorbidity. ICU admission diagnoses were led by gastrointestinal conditions (34%), followed by respiratory and “other” conditions (each 17%); smaller proportions were cardiologic (5.8%), trauma (5.8%), endocrine (5.6%), central nervous system (5.1%), infectious (4.9%), tumor (2.6%), sepsis (1.8%), and hematologic (0.2%) (Table 1).

**Table 1:** Sample characteristics.

Characteristics	n = 607
Age (Years)	64.1 (18.8)
**Gender**	
Female	276 (45%)
Male	331 (55%)
**Past medical history**	
Medically free	107 (18%)
Not medically free	500 (82%)
**ICU diagnosis**	
Cardiovascular disease	35 (5.8%)
Central nervous system	31 (5.1%)
Endocrine	34 (5.6%)
Gastrointestinal	209 (34%)
Hematology	1 (0.2%)
Infection	30 (4.9%)
Other	101 (17%)
Respiratory	105 (17%)
Sepsis	11 (1.8%)
Trauma	33 (5.8%)
Tumor	16 (2.6%)

The overall median total DDD per antimicrobial agent was 64.3 (IQR: 6.6–530.0), while the corresponding median total DOT was 47.0 (IQR: 6.0–581.5). Among the twenty-three antimicrobial agents analyzed, there was substantial variation in both the total consumption and the relative alignment between DDD-based and DOT-based indicators (Table 2). Levofloxacin and vancomycin were among the most frequently used agents, with total DDD values of 2,088.3 and 1,561.1, respectively. For these drugs, the corresponding DOT totals were 1,436 and 1,913, resulting in DDD per 100 patient-days of 31.574 and 23.602, and DOT per 100 patient-days of 21.712 and 28.923, respectively. The percentage difference for levofloxacin was 31.2%, placing it in the “major” discrepancy category, while vancomycin showed a more moderate deviation of −22.5%. Ceftriaxone and piperacillin/tazobactam exhibited close alignment between DDD and DOT, with percentage differences falling within the minor range; ciprofloxacin remained within the moderate range but showed a comparatively small discrepancy (Table 2).

**Table 2:** Comparison between DDD and DOT per 100 patient-days for the included antibiotics using the 6,614 patient-days accrued by the antimicrobial-exposed ICU cohort as the denominator.

Drug Name	DDD Total	DOT Total	DDD/100 pd	DOT/100 pd	Difference between DDD and DOT (%)	Importance of the Difference*	WHO DDD used	Mean daily dose used
**Aminoglycosides**								
Amikacin	34.55	44	0.522	0.665	−27.352	Major	1	0.775
Gentamicin	104.167	133	1.575	2.011	−27.68	Major	0.24	0.253
**Beta-Lactams**								
**Cephalosporins**								
Cefazolin	39.333	44	0.595	0.665	−11.864	Moderate	3	3.25
Ceftazidime	3.25	7	0.049	0.106	−115.385	Major	4	1.5
Ceftriaxone	370.75	357	5.606	5.398	3.709	Minor	2	2
Cefuroxime	64.25	96	0.971	1.451	−49.416	Major	3	1.875
**Carbapenems**								
Ertapenem	1	1	0.015	0.015	0	Minor	1	1
Imipenem/cilastatin	1447.7	2080	21.888	31.448	−43.676	Major	2	1.315
Meropenem	689.333	803	10.422	12.141	−16.489	Moderate	3	2.244
**Penicillins**								
Amoxicillin/clavulanate	6.25	5	0.094	0.076	20	Moderate	1.5	1.875
Piperacillin/tazobactam	1108.536	1106	16.760	16.722	0.229	Minor	14	13.414
**Cyclic lipopeptides**								
Daptomycin	6.964	5	0.105	0.076	28.205	Major	0.28	0.375
**Glycopeptides**								
Vancomycin	1561.062	1913	23.602	28.923	−22.545	Moderate	2	1.58
**Glycylcyclines**								
Tigecycline	20	7	0.302	0.106	65	Major	0.1	0.267
**Fluoroquinolones**								
Ciprofloxacin	342.7	322	5.181	4.868	6.04	Moderate	1	1.074
Levofloxacin	2088.305	1436	31.574	21.712	31.236	Major	0.5	0.758
**Lincosamides**								
Clindamycin	4	4	0.060	0.060	0	Minor	1.2	1.2
**Macrolides**								
Azithromycin	5	3	0.076	0.045	40	Major	0.3	0.5
Erythromycin	18.5	23	0.280	0.348	−24.324	Moderate	1	1.033
**Nitroimidazoles**								
Metronidazole	916.517	919	13.857	13.895	−0.271	Minor	1.5	1.483
**Polymyxins**								
Colistin	249.778	360	3.777	5.443	−44.128	Major	9 MU P; 3 MU Neb	5.05 MU/day
**Rifamycins**								
Rifaximin	84.5	47	1.278	0.711	44.379	Major	0.6	1.04
**Sulfonamides**								
Co-trimoxazole	1.939	2	0.029	0.030	−3.125	Minor	1.98	1.92

Note. DDD = defined daily dose; DOT = Days of Therapy; pd = patient-days. DDD/100 patient-days and DOT/100 patient-days were calculated using the 6,614 patient-days accrued by the antimicrobial-exposed ICU cohort as the denominator. For all non-colistin agents, WHO DDD and mean administered daily dose are shown in g/day. Colistin is shown in MU/day, using WHO route-specific DDD values of 9 MU for parenteral records and 3 MU for nebulized records. *Importance of the difference between DDD and DOT: minor (<5%), moderate (5–25%), major (>25%).

On the other hand, agents such as imipenem/cilastatin, colistin, and tigecycline demonstrated large disparities between DDD and DOT metrics. Tigecycline showed a 65.0% difference, colistin a −44.1% difference, and imipenem/cilastatin a −43.7% difference. These discrepancies were consistent with differences between the observed mean daily dose and the WHO-assigned DDD. Tigecycline had a mean daily dose of 0.267 g/day compared with the WHO DDD of 0.100 g/day, corresponding to 2.67 times the WHO DDD; its total DDD was 20.0 compared with 7 DOT. In contrast, amikacin had a mean daily dose of 0.775 g/day compared with the WHO DDD of 1.000 g/day, corresponding to 77.5% of the WHO DDD; its total DDD was 34.55 compared with 44 DOT. The direction of discordance therefore differed across agents, with DDD exceeding DOT for tigecycline and DOT exceeding DDD for amikacin.

**Fig 1:**
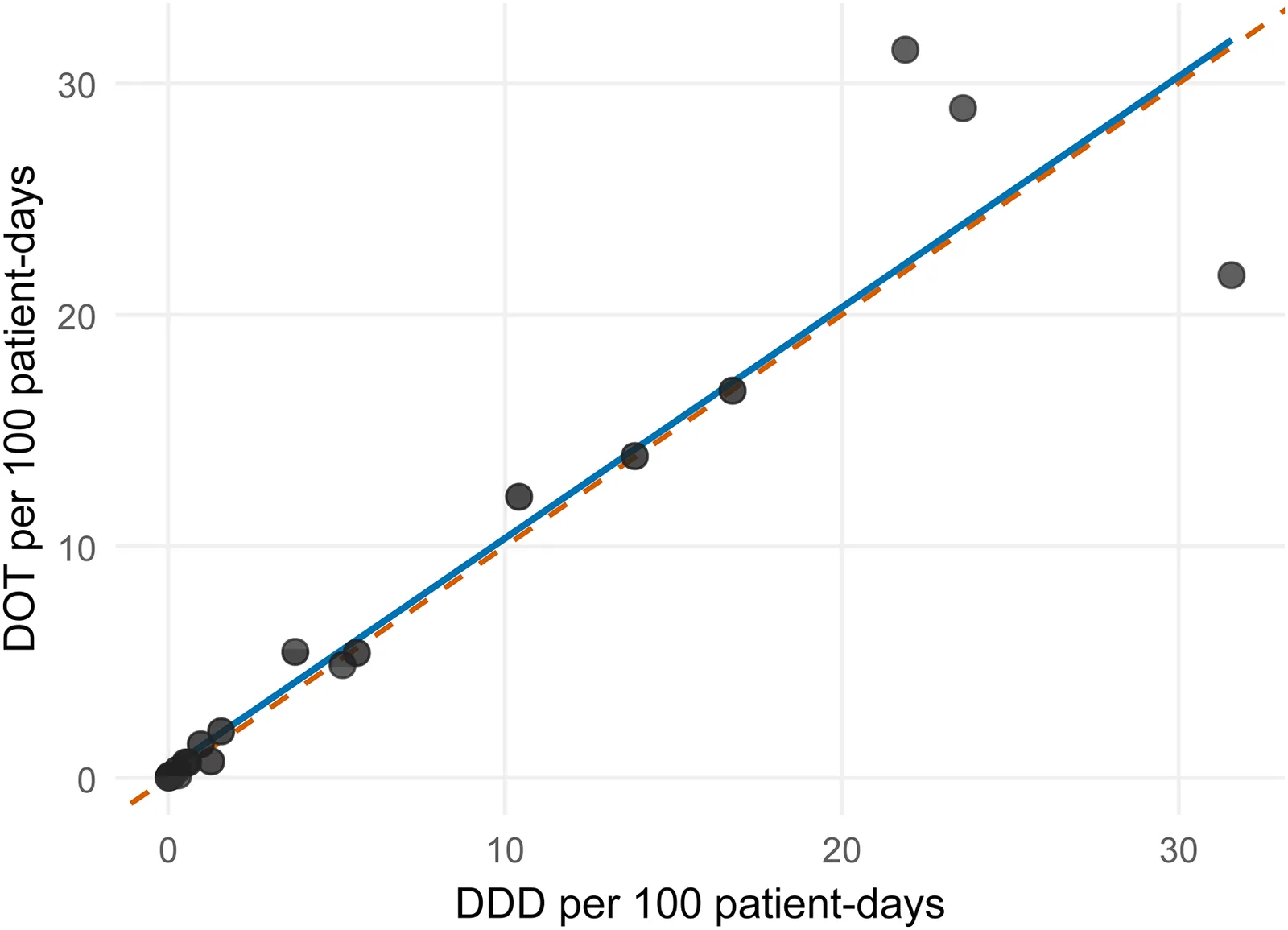
Correlation between DDD and DOT standardized per 100 patient-days. Each point represents one antimicrobial agent. The red dashed line represents the line of equality, where DDD per 100 patient-days and DOT per 100 patient-days are identical. The blue solid line represents the fitted linear regression line across antimicrobial agents.

The scatterplot of DDD versus DOT per 100 patient-days (Fig 1) displayed a broadly linear association, although a notable number of drugs diverged from the identity line. The regression analysis was performed at the drug-aggregate level (n = 23 antimicrobial agents) and indicated a strong correlation between the two metrics, with an R² value of 0.893. A Bland-Altman assessment of drug-level standardized rates showed a mean DDD-minus-DOT difference of −0.361 per 100 patient-days, with 95% limits of agreement from −6.568 to 5.847 per 100 patient-days, indicating that high correlation did not imply direct agreement for individual drugs. After repeated records for the same patient and antimicrobial were aggregated, the Wilcoxon signed-rank test comparing paired DDD and DOT totals across 1,221 patient-antimicrobial pairs remained statistically significant (V = 130,979, p < 0.001), supporting a consistent divergence between the two measures of antimicrobial exposure.

The sensitivity analysis identified two extended-interval antimicrobial records, both involving levofloxacin in one patient-antimicrobial pair. When the alternative therapeutic-coverage DOT definition was applied and values were capped at ICU length of stay, the levofloxacin DOT total increased from 1,436–1,438, and the levofloxacin DOT/100 patient-days changed from 21.712 to 21.742. The levofloxacin DDD-DOT percentage difference changed minimally from 31.236% to 31.140% and remained in the major discrepancy category. The overall findings were unchanged: the regression R² did not change meaningfully, the Bland-Altman mean difference changed from −0.361 to −0.362 per 100 patient-days, and the paired Wilcoxon comparison remained statistically significant (V = 130,820.5, p < 0.001). Detailed sensitivity results are provided in S2 Appendix.

## Discussion

In our study, we compared the DDD and DOT as metrics for measuring the amount of antibiotics consumed by 607 critically ill patients in a Jordanian ICU.

There was an absolute difference, with the overall median total DDD (64.3) being significantly higher than the median total DOT (47.0). This is consistent with similar discordance reported by Polk et al. [8], Kallen et al. [15], and Valle et al. [9]. Valle et al. emphasized that DDD is a statistical measure while DOT better reflects actual exposure in diverse populations like ICU patients.

This finding was further supported by the variation observed across the 23 antimicrobial agents, indicating that neither metric alone consistently reflects antimicrobial exposure across all drug classes. The drug-level pattern is consistent with previous antimicrobial-utilization research showing that DDD and DOT diverge when the administered daily dose differs from the WHO-assigned DDD [8,16,17]. In this dataset, close alignment for ceftriaxone and piperacillin/tazobactam contrasted with major discrepancies for levofloxacin, imipenem/cilastatin, colistin, tigecycline, rifaximin, ceftazidime, cefuroxime, and aminoglycosides. This pattern is clinically plausible because DDD is a fixed technical unit used for surveillance and benchmarking, whereas DOT reflects days of actual treatment exposure and is less affected by dose size [8].

For beta-lactams and fluoroquinolones, the size and direction of discrepancies are consistent with ICU dosing practice. Imipenem/cilastatin, ceftazidime, cefuroxime, and levofloxacin are commonly adjusted according to renal function, infection severity, and dosing interval [16]. In critically ill patients, acute kidney injury may reduce prescribed daily doses, whereas augmented renal clearance, expanded volume of distribution, severe infection, and optimized infusion strategies may lead clinicians to use regimens that differ from the WHO DDD [17]. As a result, DDD-based rates may under- or overestimate actual exposure depending on how the administered dose compares with the WHO reference dose [18].

For vancomycin and aminoglycosides, discordance is also explained by concentration-guided dosing. These drugs are frequently individualized through therapeutic drug monitoring to balance efficacy and nephrotoxicity, especially when renal function is unstable. The resulting daily dose can intentionally fall below or exceed the WHO DDD, while DOT remains tied to the number of treatment days; this explains why DOT may better preserve treatment-duration information for these agents [18,19].

Tigecycline and rifaximin illustrate a different mechanism. Tigecycline showed DDD exceeding DOT because the observed mean daily dose was 2.67 times the WHO DDD, a pattern compatible with higher-dose tigecycline strategies reported in critically ill patients with severe multidrug-resistant infections [20]. Rifaximin is indication-specific and is commonly used orally for gastrointestinal indications such as hepatic encephalopathy rather than systemic ICU infection [21]; its DDD-DOT discordance therefore reflects how a fixed WHO DDD may not capture indication-specific dosing. Colistin adds a unit-related issue because WHO DDDs are route-specific and expressed in million units rather than grams. Together, these examples support interpreting DDD as a benchmarking metric and DOT as a more clinically transparent exposure-duration metric, while recognizing that neither metric captures indication, renal function, loading doses, or therapeutic drug monitoring without additional clinical data.

There was a broadly linear relationship (R^2^ = 0.893) between DDD and DOT per 100 patient-days, even though there were differences between agents. The agreement assessment showed that this correlation should not be interpreted as interchangeability, as the 95% limits of agreement ranged from −6.568 to 5.847 DDD-minus-DOT units per 100 patient-days. A statistically significant divergence across patient-antimicrobial pairs (V = 130,979, p < 0.001) corroborated that the absolute values from DDD and DOT are not equivalent, thereby affecting reported drug consumption; a similar finding was reported by Deshwal and Tiwari [10].

Several factors may explain why DDD and DOT were strongly correlated overall but not interchangeable at the drug level. Unlike Polk et al., who reported poor correlation between aggregate DDD and DOT measures across 130 US hospitals [8], the present study was a single-center ICU analysis based on a defined antimicrobial-exposed cohort and showed a strong overall drug-level correlation. The more uniform clinical setting may have produced more consistent prescribing, dose-adjustment protocols, and antimicrobial-selection patterns than would be expected in multicenter aggregate datasets. However, consistent with Polk et al. [8], this correlation did not imply interchangeability, as several antimicrobial agents showed clinically meaningful DDD-DOT discrepancies when administered daily doses differed from WHO-assigned DDD values [9].

There are some limitations to our study. It was a single-center, retrospective study conducted in Jordan, which restricts generalizability. Future multicenter prospective studies are advised to confirm the efficacy of DOT and investigate ICU-specific metrics, taking into account distinct pharmacokinetic variations. Because the denominator comprised patient-days accrued only by the antimicrobial-exposed ICU cohort, the standardized rates are not directly comparable with whole-ICU consumption rates based on all ICU patient-days.

## Conclusion

DDD and DOT remained strongly correlated but showed clinically meaningful drug-level disagreement in this ICU cohort. DOT provides a clinically interpretable measure of antimicrobial exposure duration in ICU patients because it reflects treatment days irrespective of dose, whereas DDD remains useful for standardized benchmarking across institutions and time periods. The two measures should therefore be regarded as complementary rather than interchangeable, particularly where dose individualization, renal adjustment, therapeutic drug monitoring, or route- and unit-specific dosing may cause divergence from WHO-assigned DDD values.

## Supporting information

S1 Appendix DDD and DOT calculation reproducibility. (DOCX)

S2 Appendix Extended-interval DOT sensitivity analysis. (DOCX)
