# Supplementary material for: Evaluation of antibacterial agent use in adult patients at a tertiary-level hospital in Jordan: A comparative analysis of defined daily dose and days of therapy in critical care units
Source: PLoS One. 2026 Aug 20;21(8):e0356239. doi: 10.1371/journal.pone.0356239 (PMC13492776; doi:10.1371/journal.pone.0356239)
Supplement: Supplementary file 1 [file pone.0356239.s001.docx]

**S2 Appendix. DDD and DOT calculation reproducibility**

This appendix documents the final calculation definitions used for the DDD-DOT comparison, the denominator used for standardized rates, the analytic unit used for each statistical summary, and the verification outputs supporting Table 2 and Figure 1.

**Calculation definitions**

| **Component** | **Final definition or formula** | **Used for** |
| --- | --- | --- |
| DDD total | Total amount of drug used in the WHO-assigned unit divided by the WHO DDD for that drug and route. | Table 2, Figure 1, regression, Bland-Altman analysis, and Wilcoxon paired comparison. |
| DOT total | Number of therapy days recorded for each patient-antimicrobial record. In the primary analysis, extended-interval administrations were counted on actual administration days. | Table 2, Figure 1, regression, Bland-Altman analysis, and Wilcoxon paired comparison. |
| Standardized DDD rate | DDD/100 patient-days = (drug-level DDD total / 6,614 ICU patient-days) x 100. | Table 2 and Figure 1. |
| Standardized DOT rate | DOT/100 patient-days = (drug-level DOT total / 6,614 ICU patient-days) x 100. | Table 2 and Figure 1. |
| Percentage difference | Difference between DDD and DOT (%) = ((DDD total - DOT total) / DDD total) x 100. | Table 2 discrepancy categories. |
| Discrepancy category | Minor: absolute percentage difference <5%; moderate: 5-25%; major: >25%. | Table 2 interpretation. |
| Colistin DDD unit | Colistin was analyzed in MU, using WHO DDD values of 9 MU for parenteral records and 3 MU for nebulized records. | Colistin row in Table 2 and statistical summaries. |
| Extended-interval sensitivity analysis | Records with dosing frequency below one administration per day were re-expressed as therapeutic coverage days and capped at ICU length of stay. | S2 Appendix sensitivity analysis. |

**Analytic units**

| **Analysis/output** | **Analytic unit** | **Final n** |
| --- | --- | --- |
| Table 2 and Figure 1 | Drug-level aggregate values | 23 antimicrobial agents |
| Regression and Bland-Altman summaries | Drug-level standardized DDD and DOT rates | 23 |
| Wilcoxon signed-rank test | Patient-antimicrobial pairs after summing repeated records for the same patient and drug | 1221 |
| Sensitivity analysis | Drug-level rates and paired patient-antimicrobial values after alternative DOT counting for extended-interval records | 2 records; 1 patient-antimicrobial pair |

**R code for calculation verification**

```
library(readxl)

data_file <- "DDD_DOT_deidentified_data.xlsx"
denominator <- 6614

table2 <- read_excel(data_file, sheet = "Table2")
pairs <- read_excel(data_file, sheet = "Wilcoxon_pairs")

table2$DDD_per_100_pd_check <- table2$`DDD Total` / denominator * 100
table2$DOT_per_100_pd_check <- table2$`DOT Total` / denominator * 100
table2$percent_difference_check <- (table2$`DDD Total` - table2$`DOT Total`) / table2$`DDD Total` * 100

stopifnot(isTRUE(all.equal(table2$DDD_per_100_pd_check, table2$`DDD/100 pd`, tolerance = 1e-3)))
stopifnot(isTRUE(all.equal(table2$DOT_per_100_pd_check, table2$`DOT/100 pd`, tolerance = 1e-3)))
stopifnot(isTRUE(all.equal(table2$percent_difference_check, table2$`Difference between DDD & DOT %`, tolerance = 1e-3)))

fit <- lm(`DOT/100 pd` ~ `DDD/100 pd`, data = table2)
r_squared <- summary(fit)$r.squared
regression_p <- summary(fit)$coefficients[2, 4]

rate_difference <- table2$`DDD/100 pd` - table2$`DOT/100 pd`
bland_altman <- data.frame(
  mean_difference = mean(rate_difference),
  lower_limit = mean(rate_difference) - 1.96 * sd(rate_difference),
  upper_limit = mean(rate_difference) + 1.96 * sd(rate_difference)
)

wilcoxon_test <- wilcox.test(
  pairs$DDD_total_pair,
  pairs$DOT_total_pair,
  paired = TRUE,
  exact = FALSE,
  correct = FALSE
)

list(
  regression_r_squared = r_squared,
  regression_p_value = regression_p,
  bland_altman = bland_altman,
  wilcoxon_n = nrow(pairs),
  wilcoxon_V = unname(wilcoxon_test$statistic),
  wilcoxon_p_value = wilcoxon_test$p.value
)
```
