# Supplementary material for: Evaluation of antibacterial agent use in adult patients at a tertiary-level hospital in Jordan: A comparative analysis of defined daily dose and days of therapy in critical care units
Source: PLoS One. 2026 Aug 20;21(8):e0356239. doi: 10.1371/journal.pone.0356239 (PMC13492776; doi:10.1371/journal.pone.0356239)
Supplement: Supplementary file 2 [file pone.0356239.s002.docx]

**S2 Appendix. Extended-interval DOT sensitivity analysis**

This appendix summarizes the sensitivity analysis evaluating whether counting extended-interval dosing as therapeutic coverage days, rather than administration days only, changes the DDD-DOT comparison.

The dataset contained 2 extended-interval antimicrobial records, all involving levofloxacin and representing 1 patient-antimicrobial pair.

| **Metric** | **Primary analysis** | **Sensitivity analysis** | **Interpretation** |
| --- | --- | --- | --- |
| Affected antimicrobial | levofloxacin | levofloxacin | No other antimicrobial had extended-interval records. |
| Affected records | 2 | 2 | Two records from one patient-antimicrobial pair. |
| Levofloxacin DOT total used for Table 2/Figure 1 | 1436 | 1438 | Increase of 2 DOT. |
| Levofloxacin DOT/100 patient-days | 21.712 | 21.742 | Negligible change in standardized rate. |
| Levofloxacin DDD-DOT percentage difference | 31.236% | 31.140% | Remained in the major discrepancy category. |
| Regression R-squared | 0.893336 | 0.893680 | No meaningful change. |
| Bland-Altman mean difference | -0.361 | -0.362 | No meaningful change. |
| Bland-Altman 95% limits of agreement | -6.568 to 5.847 | -6.561 to 5.837 | Limits were effectively unchanged. |
| Wilcoxon signed-rank test | V = 130,979; p <0.001 | V = 130,820.5; p <0.001 | The paired comparison remained statistically significant. |

Note. The sensitivity analysis counted therapeutic coverage days for extended-interval records and capped patient-antimicrobial DOT values at ICU length of stay. Table 2 and Figure 1 use drug-level totals. The Wilcoxon signed-rank test uses paired patient-antimicrobial records with complete DDD and DOT values; in that paired dataset, levofloxacin DOT changed from 1,435 to 1,437.
